# Listening in complex acoustic scenes

**DOI:** 10.1016/j.cophys.2020.09.001

**Published:** 2020-09-08

**Authors:** Andrew J King, Kerry MM Walker

**Affiliations:** Department of Physiology, Anatomy and Genetics, University of Oxford, Oxford OX1 3PT, UK

## Abstract

Being able to pick out particular sounds, such as speech, against a background of other sounds represents one of the key tasks performed by the auditory system. Understanding how this happens is important because speech recognition in noise is particularly challenging for older listeners and for people with hearing impairments. Central to this ability is the capacity of neurons to adapt to the statistics of sounds reaching the ears, which helps to generate noise-tolerant representations of sounds in the brain. In more complex auditory scenes, such as a cocktail party — where the background noise comprises other voices, sound features associated with each source have to be grouped together and segregated from those belonging to other sources. This depends on precise temporal coding and modulation of cortical response properties when attending to a particular speaker in a multi-talker environment. Furthermore, the neural processing underlying auditory scene analysis is shaped by experience over multiple timescales.

## Introduction

Most research on the auditory system focuses on the way single sound sources are processed and perceived. In real life, however, the sounds reaching our ears usually comprise a mixture of signals arising from multiple sources. A major challenge faced by the auditory system is therefore to group together the sound attributes associated with a particular source and segregate them from those belonging to other sources. This is auditory scene analysis [1 ^•^]. In order to follow a conversation in a busy restaurant, for example, the brain has to be able to separate the voice of the person speaking to you from the babble of other, superimposed voices that overlap in time. This ‘cocktail party problem’ [2,3] represents a particular challenge for the auditory system since each of the voices will likely resemble one another both acoustically and perceptually.

**Figure 1:**
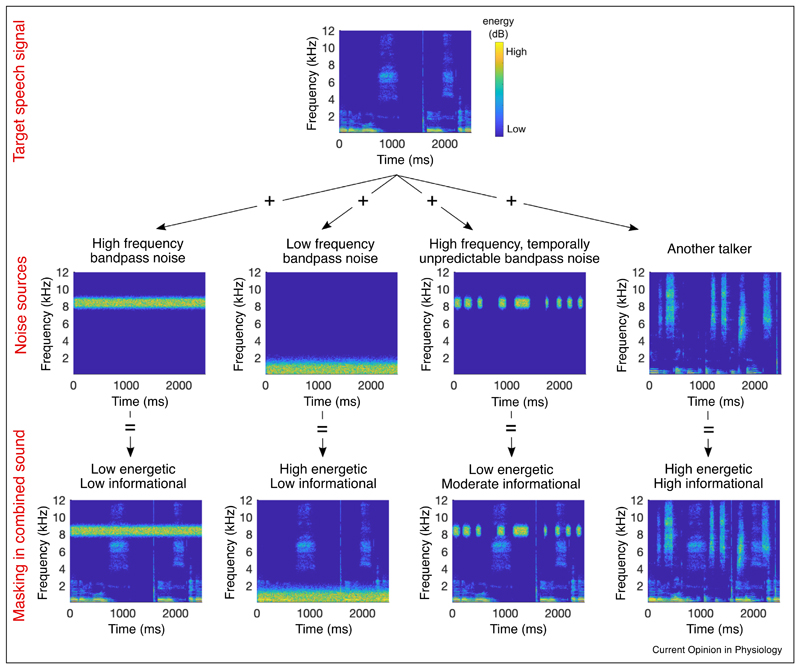
Energetic and informational masking of speech. The spectrogram of a speech stream of interest is shown in the top panel. The spectrograms of four example noise sources are shown in the middle row. Spectrograms of the mixtures of the target speech stream and each noise are shown in the bottom row. A steady-state, high-frequency bandpass noise provides less energetic masking of the speech (column 1) than a similar noise with a lower frequency band (column 2), as most of the energy in the speech is low frequency. Reducing the temporal predictability of the noise increases the informational masking (column 3), as the listener’s attention is captured by the noise and the auditory system cannot as easily adapt to it. The unpredictable noise will therefore be more disruptive to speech intelligibility even though it provides less energetic masking than the noise sources in the two left columns. Finally, the speech of a second person talking provides high amounts of energetic and informational masking (column 4), making it more difficult to segregate and attend to the target speech.

There are two reasons why the presence of other voices may make it difficult to pick out the speech signal of interest [4,5]. First, due to their overlapping spectra, the voices compete to activate the same frequency channels in the auditory system, which is known as energetic masking. This reduces the audibility of the target voice by weakening its representation in both the cochlea and the central auditory pathway. Second, competing voices may result in informational masking, drawing the listener’s attention away from the target voice. The unpredictability of the background voices and shared higher-level statistical features with the target speaker impede our attempts to ignore them, introducing perceptual uncertainty as to what that speaker was saying (Figure 1).

The brain’s solution to this problem is theorized to be composed of at least two processes, which are not entirely independent [1 ^•^,^3^]. First, the features of the various sounds in the environment that reach the ear as a mixture need to be extracted and segregated into those that correspond to different sound sources. In this manner, the features of the target sound are bound together into a single perceived object, such as a person’s voice. Second, there must exist a mechanism for attending to the bound features of the target sound source of interest, moving other auditory information into the perceptual background.

While cocktail parties represent a particularly challenging situation for the auditory system, most behaviorally important sounds are heard against a background of everyday noise — from street and workplace sounds to music — which therefore represents a simpler example of auditory scene analysis. Understanding the neural basis for listening in noise has considerable clinical and societal relevance since many people experience difficulties with this vital task. This is particularly the case with increasing age [6] and in individuals with hearing impairments, even when amplification is provided to compensate for raised thresholds [7]. But irrespective of age, people with audio-grams within the normal range can show marked differences in speech-in-noise performance [8]. This has been attributed to physiological differences in the way individuals process the temporal structure of sounds [8,9], and in their ability to attend to specific speech streams [9,10] or group sound elements as belonging to foreground or background sounds [11]. Although cochlear abnormalities that do not show up in the audiogram are likely to contribute too [12], these findings indicate that impairments of listening in noise at least partially reflect a deficit in central auditory processing.

## Adaptation and the background noise problem

An effective solution has evolved for coping with the challenge of hearing in noisy environments, at least when the statistics of the foreground and background sounds differ. A key feature of sensory neurons is that they can adapt their responses to match these statistics. If the frequency content and level of the background noise vary relatively slowly compared to that of the human voice or other sounds of interest, neuronal adaptation can serve to reduce neuronal responses to the noise and therefore improve the audibility of the target sound [13].

### Efferent control of the cochlea

Evidence for adaptation to background noise has been obtained in studies of human hearing, since speech-in-noise recognition improves if the masking noise starts before the speech sounds rather than at the same time [14,15]. Because they predominantly innervate outer hair cells in the contralateral cochlea, medial olivocochlear neurons in the superior olivary complex have long been implicated in enhancing sound detection in background noise [16,17]. However, recent studies in which noise adaptation was reduced by introducing fluctuations into the noise [18] and of noise adaptation during word recognition in cochlear implant users [19], in whom the medial olivocochlear reflex is not thought to operate, has cast doubt on this. Furthermore, measurements of otoacoustic emissions indicate that post-adaptation improvements in sensitivity to amplitude modulation for tones presented in noise are unlikely to be due to an efferent-dependent reduction in cochlear responses [20]. Nevertheless, through their influence on outer hair cells, medial olivocochlear efferents can regulate cochlear gain and therefore the responses of auditory nerve fibers, and their role in listening in noise remains a controversial area [21,22].

### Neuronal adaptation to sound level and contrast

In the last few years, investigation of the neurophysiological basis for noise adaptation has focused primarily on central auditory processing. Adaptation to sound level statistics is found throughout the auditory system from the auditory nerve to the cortex [23–27]. The dynamic range — the range of stimulus values encoded by a neuron by a change in its firing rate — can shift to compensate for changes in mean stimulus level, thereby maintaining maximum sensitivity over the most commonly encountered values. In addition to changing the mean overall sound level, the presence of background noise will alter the contrast, that is the variance in the sound level distribution [13]. Again, the brain can adapt to this by scaling neuronal response gain to compensate for changes in stimulus contrast. Contrast gain control is also a common property of neurons along the auditory pathway [28,29 ^•^,^30^,^31^^•^].

**Figure 2:**
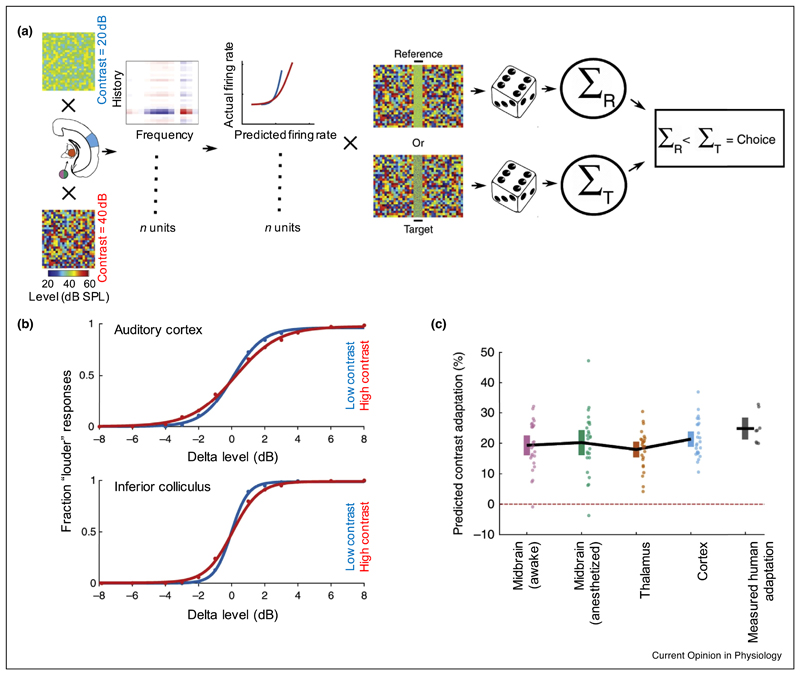
The strength of perceptual contrast adaptation can be predicted from the contrast adaptation exhibited by auditory neurons. **(a)** Schematic of a computational model that uses neuronal responses recorded from neurons in the mouse auditory midbrain, thalamus or cortex to predict performance on a 2-alternative, forced-choice sound level discrimination task for pairs of broadband noise bursts presented in different contrast environments. Simulated responses to noise stimuli of different levels (reference, R: 70 dB SPL, target, T: 62–78 dB SPL), embedded in low- or high-contrast dynamic random chords, were derived from the contrast-dependent linear/non-linear model estimated from the individual neuronal responses recorded at each processing level. Psychometric functions were determined by asking which noise stimulus elicited most spikes across all recorded units in the simulated trial. If the reference noise elicited fewer spikes than the target noise stimulus a “louder” response was predicted. See Lohse et al. [31 ^•^] for more details. **(b)** Psychometric functions produced by the model based on responses recorded in the primary auditory cortex (top) or in the midbrain from the central nucleus of the inferior colliculus (bottom) in low-contrast (20 dB, blue) and high-contrast (40 dB, red) conditions. Level discrimination improved when the contrast of the flanking sounds was low, as indicated by the steeper psychometric functions. **(c)** Predicted strength of contrast adaptation from neuronal responses recorded in the auditory midbrain of awake mice or in the midbrain, thalamus or cortex under anesthesia, compared with measured perceptual contrast adaptation in human listeners. Note the similarity in each case. Solid black lines connect mean values after 25 runs of the model (or across the eight participants in the measured human adaptation). Colored error bars denote 95% confidence intervals around the mean (for clarity, individual data points are displayed next to the corresponding error bars). Adapted from Lohse *et al*. [31 ^•^].

The functional consequences of adaptation to sound statistics are still relatively unexplored. In monkeys, adaptation of inferior colliculus (IC) neurons to mean sound level does not appear to affect their neurometric thresholds or the animals’ psychometric thresholds for tones presented in noise [32]. On the other hand, dynamic range adaptation is associated with perceptual adjustments in human spatial hearing [33,34]. Moreover, sound level discrimination thresholds in human listeners vary with stimulus contrast and the strength of this perceptual adaptation can be predicted from the contrast gain control exhibited by neurons at both subcortical and cortical levels in the mouse [31 ^•^] (Figure 2).

## Noise-tolerant coding of sounds in the auditory cortex

**Figure 3:**
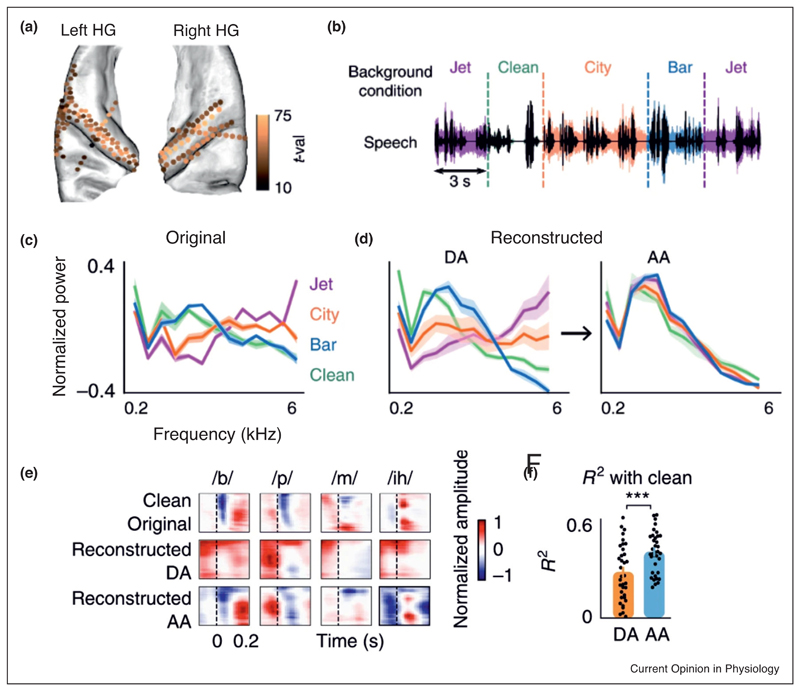
Adaptation of the human auditory cortex to changing background noise enables robust representation of the phonetic features of speech. **(a)** Recordings in human auditory cortex showing electrode locations where significant responses to speech (t-val > 10, *t*-test speech versus silence) were found. HG, Heschl’s gyrus. **(b)** Waveforms of the sounds used: speech (shown in black) was presented alone (Clean) or with different types of background noise (shown by the colors for Bar, City, Jet backgrounds), which changed randomly every 3 or 6 s. **(c)** Average frequency power from the spectrograms for each stimulus type. **(d)** A reconstruction model was trained on the responses to clean speech and used to reconstruct spectrograms from the neural responses to speech with added background noise. Left panel shows the average reconstructed frequency profiles during adaptation (DA), which resemble the frequency profiles for each noise type. Right panel shows that after adaptation (AA) of cortical responses, the average reconstructed frequency profile in each case closely resembles the frequency profile of clean speech. **(e)** Original and reconstructed spectrograms of four example phonemes. The spectrotemporal features that distinguish each of these phonemes in the spectrograms reconstructed from cortical activity are initially distorted during adaptation to the noise (0–0.4 s after the change in stimulus), but are evident after adaptation (2.0–2.4 s after the transition). For example, the phoneme /*b*/ is characterized by an onset gap followed by low-frequency spectral power. Both the gap and the low-frequency feature are masked during adaptation, but are subsequently restored after adaptation. **(f)** Correlation between the reconstructed phoneme spectrograms during and after adaptation with the clean phoneme spectrograms. Adapted from Khalighinejad *et al*. [37 ^••^].

Several studies have shown that adaptive coding gradually builds up along the auditory pathway [25,29 ^•^,^31^^•^]. As a consequence, by the level of the primary auditory cortex (A1), adaptation to mean level and contrast enables speech to be represented in a way that is relatively robust to the presence of stationary background noise [29 ^•^]. Other studies have also reported a role for adaptation in generating noise-tolerant cortical representations of speech [35,36,37 ^••^]. For example, electrocorticography (ECoG) data obtained from neurosurgical patients listening to speech in the presence of abruptly changing background noise have shown that auditory cortical neurons rapidly adapt to the noise, resulting in enhanced neural coding and perception of the phonetic features of speech [37 ^••^] (Figure 3). Furthermore, fMRI responses to natural sounds presented in isolation and in real-world noise are more noise invariant in non-primary auditory cortex than in primary areas [38].

How stimulus processing changes in the presence of noise is actually not straightforward. The effects of noise on frequency selectivity in rat A1 and on word recognition performance in humans depend not only on the signal-to-noise ratio, but also on the absolute levels of the foreground tones and background noise [39]. Moreover, cortical neurons differ in how accurately they encode target stimuli in the presence of noise [40]. Surprisingly, the presence of continuous broadband noise has been found to improve tone discrimination for small frequency differences in mice and this behavioral improvement could be replicated by optogenetically activating parvalbumin-positive interneurons in order to make A1 tuning curves resemble those recorded in the presence of noise [41 ^•^]. Furthermore, A1 neuronal sensitivity to tones presented in noise is enhanced if coherently modulated sidebands are added to the noise masker, a condition that improves signal detection thresholds in humans [42]. Like the release from masking found for speech-in-noise recognition noise by human listeners [14,15], prior adaptation to the noise is required to produce substantial comodulation masking release in A1 and this was reduced by inhibiting cortical activity during the priming period [42].

Given the importance of the cortex in listening in noise, attention is turning to the cellular circuits and synaptic mechanisms responsible for the adaptive processing of auditory information [43,44]. Nevertheless, we should not ignore what happens at subcortical levels. Although recent work in guinea pigs has confirmed that cortical neurons are more tolerant to changes in noise level, populations of neurons in the medial geniculate body (MGB) and particularly the IC actually show greater discrimination performance for communication calls presented in noise than those recorded in the cortex [45]. It is possible that descending corticofugal projections contribute to context-dependent processing at subcortical levels. Neither adaptation to mean level by IC neurons [26] nor to contrast by IC or MGB neurons [31 ^•^] depends on corticofugal inputs, but cortical inactivation does slow down sound level adaptation by IC neurons when the same sound statistics are re-encountered [26]. This suggests that descending projections might play a role in adaptation to stimulus statistics in rapidly changing acoustic environments.

## Environmental statistics and scene analysis

The brain is faced with the challenge of not only identifying different sources from the mixture of sounds reaching the ears, but also of separating those sources from environmental information that may be present too. Within rooms and other enclosed spaces, sound arrives directly from its source, accompanied by multiple delayed versions due to reflections off the walls and other surfaces. Reverberation is useful because it helps the listener to estimate room size [46] and source distance [47], but it also distorts the sound arriving from the source. However, listeners can perceptually separate sound sources and the accompanying reverberation, though this ability is impaired if the environmental statistics deviate markedly from natural values measured in a range of inner-city and rural locations [48 ^•^]. This study therefore suggests that prior experience of these statistics is important for perception.

## Sound source segregation and selective attention

Listening to speech in the presence of a constant noise source, like an airplane passing overhead, is challenging, but is aided by the way neurons adapt to low-level statistics, like sound level and contrast. However, it is considerably more difficult to filter out less predictable noise sources, such as background speech, because they provide more informational masking [49].

Several ‘primitive’ perceptual features, which can be derived from the bottom-up stimulus statistics of most sounds, have been shown to contribute to source segregation. These include differences in the level, spatial location, timbre (e.g. the spectral envelopes), temporal modulation and harmonicity of the sounds produced by separate sources [1 ^•^,^3^]. Each of these features is extracted through specialized mechanisms throughout the ascending auditory system, which are beyond the scope of this review. Individual neurons in ferret A1 can represent multiple features by multiplexing several neural codes [50], and recent evidence suggests that this strategy may also be employed in human auditory cortex [51,52]. When multiplexing cortical neurons are co-activated by several features of a single sound source, they may bind these features through their simultaneous activity within the wider cortical network, consistent with predictions of the temporal coherence theory of auditory scene analysis [53].

Neural synchronization may also help to bind coincident features across sensory systems. The addition of temporally coherent visual cues can enhance the representation of a target speech stream in auditory cortex [54], and this has been shown to be mediated by visual cortex [55 ^•^]. These neural processes may account for why face reading helps human listeners to selectively attend to a single speaker in multi-talker listening situations [56].

The process of grouping sound features belonging to a single source depends critically upon encoding their onsets and offsets [53,57]. The precise representation of the timing of acoustical events in the auditory system is well suited to provide this essential information. Accurate neural representations of sound offsets are generated through specialized post-inhibitory rebound mechanisms in the dorsal cochlear nucleus [58] and superior paraolivary nucleus [59], and remain temporally precise in regions of the MGB [60]. These thalamic neurons in turn form offset-encoding synapses onto auditory cortex neurons that appear to be distinct from those representing sound onsets [61]. The auditory cortex also makes use of local inhibitory inputs to sharpen temporal spiking responses [62,63]. Furthermore, Fishbach *et al.* [64] have shown that A1 neurons produce enhanced responses to feature onsets, which may highlight the beginning of an auditory event in complex scenes.

Many natural sounds, including voices, are composed of frequencies that are harmonics of a common fundamental frequency. Such sounds are perceived as a single auditory object with a pitch at the fundamental frequency. Therefore, harmonicity is a useful cue for grouping sound sources in busy auditory scenes, including multi-talker environments [65 ^••^]. Harmonic grouping cues are often examined experimentally by mistuning one tone of a harmonic tone complex, which results in perception of the mistuned tone as a separate auditory object (e.g. Ref. [66]). Human [67] and macaque [68] auditory cortex produce enhanced responses to tone complexes that contain a mistuned harmonic, which correlate with subjects’ perception of a second auditory source [67]. Descending feedback from cortex may play a key role in the process of harmonic scene segregation. Deactivating the auditory cortex disrupts the neural representation of the relative levels of two concurrent harmonic sounds in the IC [69], while lesioning the connections from A1 to the MGB impairs ferrets’ performance on a mistuning detection task [70 ^•^].

## Sensory experience and scene analysis

Listeners can learn to group known feature combinations, or use new statistical features, in order to better segregate sound sources within their individual auditory environments. In a process described by Bregman as ‘schema-based integration’ [1 ^•^], a listener can rely on a particular combination of sound features to segment a familiar sound source, be it the sound of their spouse’s voice [71], a familiar language [72], or a statistical regularity encountered during an experiment [73]. The latter study demonstrated that schema learning can be rapid and implicit, and is derived from the statistics of the current acoustic environment [73]. Młynarski and McDermott [74] further showed that grouping is not limited to the well-studied primitive grouping features (harmonicity, common onsets and offsets, etc), but is also based on previously unexplored spectrotemporal features that commonly co-occur in natural sounds, such as speech and music. Thus, the features used for auditory scene analysis are more numerous and varied than previously appreciated.

Prior exposure to the target speech stream facilitates speech segregation in a multi-talker listening task, and magnetoencephalographic (MEG) recordings have shown that this principally involves reduced tracking in the auditory cortex of the non-primed speech stream [75]. Thus, auditory scene analysis is influenced by the statistical properties of the input, as well by linguistic information held in working memory. However, the neural basis by which we flexibly derive and use grouping features to segregate sounds is largely unexplored, particularly at the cellular level.

As mentioned in the introductory section of this review, the ability of listeners to understand speech in the presence of other sounds varies between individuals, even when factors like age and hearing status are taken into account [8,10]. Musical experience is likely to play a role here [6,8], and various forms of training have been shown to be effective in improving speech-in-noise perception [76,77 ^•^]. Furthermore, raising rats in the presence of noise with various spectrotemporal statistics leads to enhanced behavioral performance and A1 encoding of vocalizations in noise [78 ^••^]. Collectively, these studies highlight the importance of experience in shaping the capacity of the brain to segregate sound sources.

## Cortical correlates of attending to a single talker

**Figure 4:**
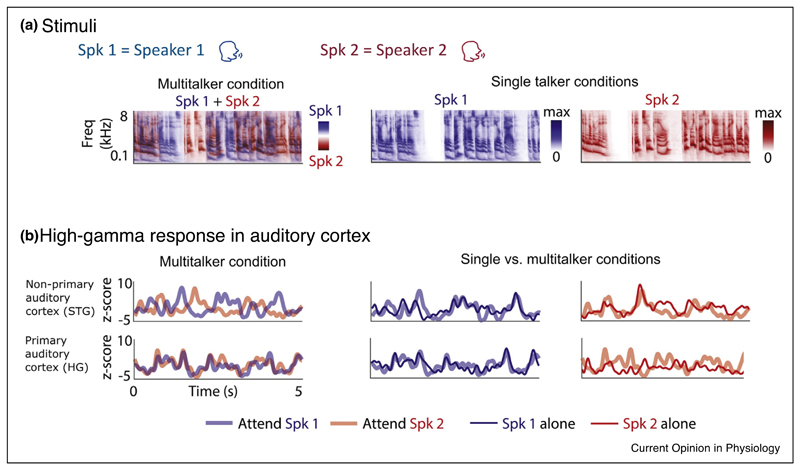
Representations of attended and unattended speech in human auditory cortex. **(a)** Each plot shows a spectrogram of speech presented to listeners, produced by speaker 1 (Spk 1; male; shown in blue) or speaker 2 (Spk 2; female; shown in red). The left panel is a spectrogram of speech from both speakers when presented together. The spectrotemporal content of the two speech streams largely overlaps, as in many real-world environments. The same speech streams presented in isolation are shown in the spectrograms on the right. **(b)** Example high-gamma responses from two depth electrodes recorded in one subject: one in the non-primary auditory cortex (superior temporal gyrus; STG) and the other in primary auditory cortex (Heschl’s gyrus; HG). The response in the non-primary auditory cortex changes depending on which speaker is being attended, and resembles the response to that speaker in isolation. Conversely, the response in primary auditory cortex is similar when attending to either speaker, and is dominated by the spectrotemporal content of speaker 1. Therefore, responses in the primary auditory cortex are determined by the spectrotemporal content of the speech, irrespective of attention, whereas responses in non-primary auditory cortex better represent the attended speaker. Adapted from O’Sullivan *et al*. [82 ^••^].

Once features from different sound sources are segmented, we can guide our attention to a single source of interest. Studies throughout the past decade have substantially improved our understanding of how selectively listening to a single speaker in a multi-talker environment alters cortical representations of sounds. Studies using MEG [79], EEG [80], ECoG arrays [81,82 ^••^] and depth electrodes [82 ^••^] have described an enhanced representation of the attended speech in human auditory cortex during these selective attention tasks (Figure 4). In fact, the attended speech stream dominates the cortical response to the extent that it can be accurately reconstructed from neural responses to the sound mixture as if it were presented alone [81,82 ^••^]. Several studies suggest that this form of attentional enhancement is observed in secondary, but not primary, auditory cortex [79,82 ^••^,^83^]. This is indicated by the late (>100 ms) timing of attentional effects that have been reported in MEG [79], EEG [80] and ECoG [81,82 ^••^] studies, even if the two speech streams are presented to different ears, providing a low-level binaural cue [84]. Studies using depth electrodes [82 ^••^] and fMRI [85] further support the functional localization of these atten-tional effects to the superior temporal gyrus.

The cellular mechanisms giving rise to selective listening effects in the presence of high energetic and informational masking remain largely unexplored in animal models. Because two competing speech streams often overlap substantially in their frequency content and simple stimulus statistics, the neural processes are likely to act on higher-order perceptual features (such as voice timbre and pitch [86]), which simple frequency gain filters alone are insufficient to explain.

## Applications beyond sensory neuroscience

Our growing understanding of the neurophysiology of auditory scene analysis has important applications in artificial intelligence and clinical practice. The budding field of computational auditory scene analysis draws inspiration from neural algorithms to improve automatic speech recognition [87]. In another promising area of research, Han *et al.* [88] have demonstrated that the acoustics of an attended auditory source can be recovered online by measuring and decoding its enhanced representation in auditory cortex — even without knowledge of how the voices sound in isolation. The hope is that this information can be used to amplify target-relevant acoustical features in a listener’s hearing prosthetic within a crowded auditory scene. This essentially involves reading the answer to the cocktail party problem from the brain itself, and feeding it back into the listener’s hearing aid. As our knowledge of the biological solutions to the cocktail party problem improve, so will our ability to support hearing and communication throughout people’s lifespans.
